# Bajan Birds Pull Strings: Two Wild Antillean Species Enter the Select Club of String-Pullers

**DOI:** 10.1371/journal.pone.0156112

**Published:** 2016-08-17

**Authors:** Jean-Nicolas Audet, Simon Ducatez, Louis Lefebvre

**Affiliations:** Department of Biology, McGill University, Montréal, Québec, Canada; Consiglio Nazionale delle Ricerche, ITALY

## Abstract

String-pulling is one of the most popular tests in animal cognition because of its apparent complexity, and of its potential to be applied to very different taxa. In birds, the basic procedure involves a food reward, suspended from a perch by a string, which can be reached by a series of coordinated pulling actions with the beak and holding actions of the pulled lengths of string with the foot. The taxonomic distribution of species that pass the test includes several corvids, parrots and parids, but in other families, data are much spottier and the number of individuals per species that succeed is often low. To date, the association between string-pulling ability and other cognitive traits was never tested. It is generally assumed that string-pulling is a complex form of problem-solving, suggesting that performance on string-pulling and other problem-solving tasks should be correlated. Here, we show that individuals of two innovative species from Barbados, the bullfinch *Loxigilla barbadensis* and the Carib grackle *Quiscalus lugubris fortirostris*, pass the string-pulling test. Eighteen of the 42 bullfinches tested succeeded, allowing us to correlate performance on this test to that on several other behavioral measurements. Surprisingly, string-pulling in bullfinches was unrelated to shyness, neophobia, problem-solving, discrimination and reversal learning performance. Only two of 31 grackles tested succeeded, precluding correlational analyses with other measures but still, the two successful birds largely differed in their other behavioral traits.

## Introduction

String pulling behavior was first described nearly two millennia ago by Pliny the Elder, who observed captive goldfinches pulling buckets of water (see [1]). Since then, the string-pulling task and its numerous variations have been used on several taxa such as cats [2], dogs [3] and apes [4]. Still, cognitive studies involving birds dominate the string pulling literature [1]. In birds, the paradigm involves retrieving a visible, out-of-reach reward by pulling a vertical string that is sufficiently long so that the bird has to pull sequentially several times while maintaining the pulled portions of the string with its foot. String pulling is considered one of the most complex problem-solving tasks and it has been proposed that “insight” [5–7] or “imagination” [8] are required as the test is quickly passed by some animals without any apparent trial-and-error [7]. However, such explanations have been the focus of much debate and most researchers, on the basis of tests that disrupt direct string-reward connections, agree nowadays that animals use positive perceptual-motor feedback as a reinforcement to eventually complete the task [1,3,9–14]. Whatever the case may be, completing this task involves a degree of sequential coordination, as it requires successive actions that are not immediately rewarded, where a bird has to produce a coherent suite of pulling movements with its beak and holding loops of string with its feet.

Although the number of bird species shown to solve the string-pulling task is growing steadily, most of them belong to only a restricted set of families. Large-brained species from the parrot and Corvid clades make up the vast majority of them, while tests on several *Paridae* (tits) species also yield positive data (see Table 3 in [1]). Studies on other Oscine families reveal much more mixed results. The avian superfamily *Passeroidea* in particular, an extremely diverse and globally distributed clade, shows wide variation between species and individuals in success or failure at the test. Within *Fringillidae*, for example, the family that includes the best-studied species, the Eurasian goldfinch, eight out of the ten species listed in [1] yield both positive and negative results. Outside of Corvids, Psittacids and Parids, there is thus extensive taxonomic and individual variation. Testing new species is important to obtain a coherent picture of taxonomic variation if we are to compare string-pulling to other, well-studied cognitive measures. In particular, associations between different cognitive measurements and performance at string pulling could provide information on the abilities required to succeed at this task. In addition, the string-pulling paradigm is one of a very few tasks that can be used across a wide variety of taxa.

Two other features of the avian string-pulling literature research are also noteworthy. First, most birds used in the studies were raised in captivity, be it in laboratories or zoos. Familiarity with (and often hand-raising by) humans might facilitate solving of the string problem, with, for example, conspecific tutoring [11] or long periods of acclimatization [15] sometimes included in the protocol. Notable exceptions are the work of Taylor and colleagues [12,13] on wild-caught crows *Corvus moneduloides* studied in aviaries in their native New Caledonia, as well as that of Millikan and Bowman [16] on seven species of Darwin's finches. Second, several studies were done on a very small number of individuals per species or a number that is unspecified in the papers. Given the often low success rate obtained on species where large numbers of individuals are tested, false negatives might be frequent when species with small sample sizes fail the test.

In this paper, we test wild-caught individuals from two species not previously examined and attempt to correlate their string-pulling performance with their results on other tests. We show that wild-caught Barbados bullfinches *Loxigilla barbadensis* and Carib grackles *Quiscalus lugubris fortirostris*, two innovative Barbadian species [17], pass the basic string-pulling test, and we ask if individual variation in performance can be predicted by results on other tasks. From previous experiments on the same individuals [18,19], we had data on shyness, neophobia, problem-solving, discrimination learning and reversal learning. The shyness test assessed an individual's latency to feed after being disturbed by an experimenter, while neophobia measured a similar latency when a novel object was placed near the food. Two problem-solving tasks required novel motor acts to access visible food. In the discrimination-learning task, individuals needed to choose the correct color cue identifying a food container. In the reversal learning test, individuals needed to reverse their former association by inhibiting responses towards the formerly rewarded color, shift their attention and form a new association with the previously unrewarded color. Based on previous results on the same species, we predicted that string-pulling would correlate with the other problem-solving tasks, but would show either a negative or no correlation with discrimination learning and/or reversal scores [18,19]. Several studies suggest that discrimination and reversal learning paradigms measure different abilities compared to problem-solving tasks [18–22].

Millikan and Bowman [16] have previously tested species from the *Passeroidea* superfamily to which our grackles (family *Icteridae*) and Barbados bullfinches (family *Thraupidae*) belong. Neither of the two Icterids (Brewer’s blackbird, *Euphagus cyanocephalus* and Red-winged blackbird, *Agelaius phoeniceus*) passed the test, nor did the only Thraupid that is not a Darwin's finch, the Cuban grassquit *Tiaris canora*, a close relative of the Barbados bullfinch [23]. Given that the number of individuals tested per species is not mentioned in Millikan and Bowman [16], these results could be false negatives if sample sizes were small. Here, we test a total of 73 wild-caught birds and show that individuals from our two species can pass the string-pulling test.

## Methods

### Subjects

All birds were captured between February and April 2013 and were kept in individual cages at the Bellairs Research Institute, St. James, Barbados. Details on captures and housing conditions are given in [18], [19], and in the S1 Appendix). Briefly, forty-two Barbados bullfinches were caught at eight different sites on the island of Barbados, and thirty-one Carib grackles on the grounds of the Bellairs Research Institute of McGill University, St. James, Barbados. All birds were released at their initial site of capture at the end of our experiments. All our procedures were approved by the McGill University Animal Care Committee (Animal Use Protocol 2013–7140) as well as the Natural Heritage Department of the Barbados Ministry of Environment and Drainage (permit 8434/56).

### Experimental procedures

**Fig 1:**
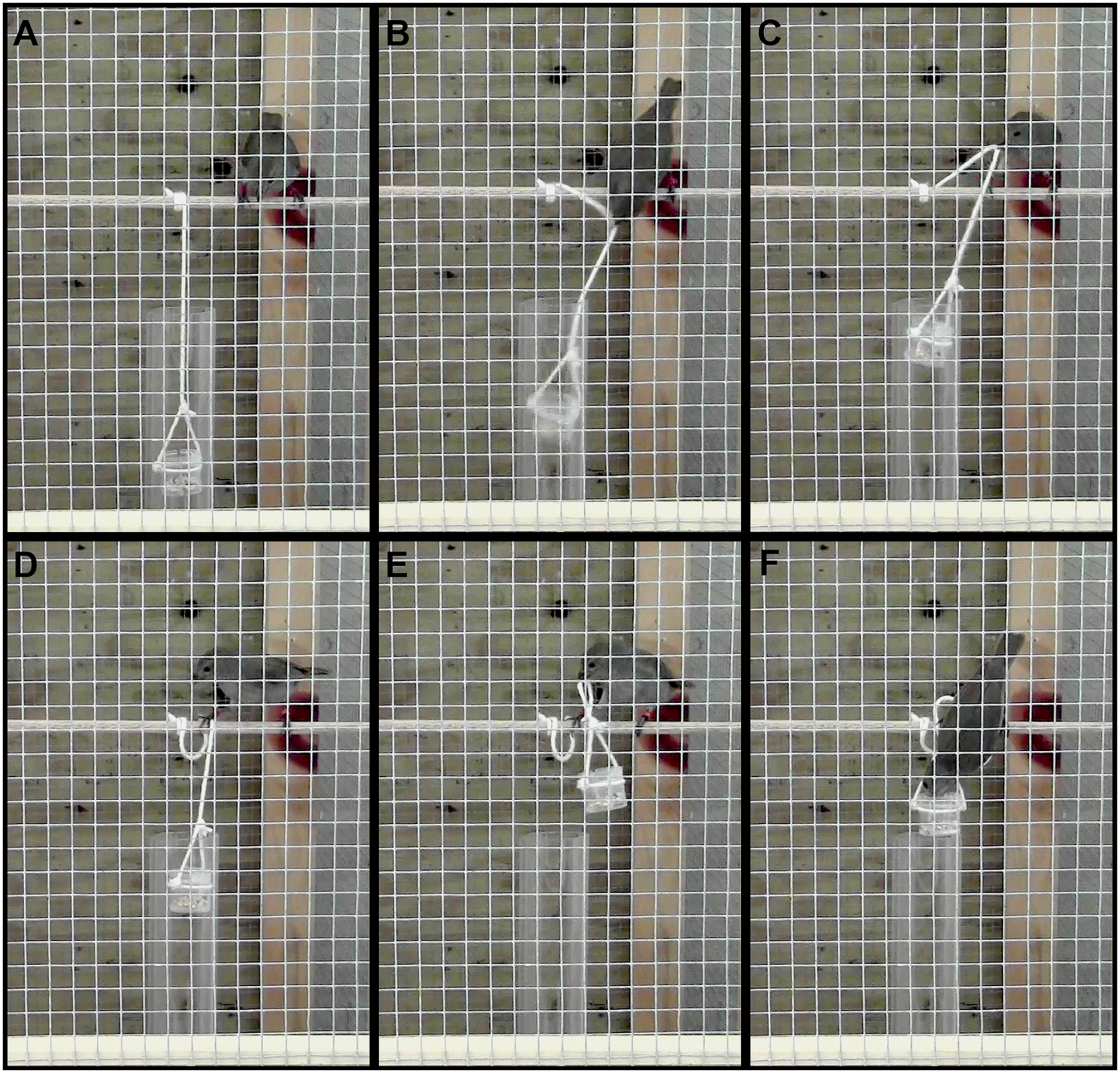
Typical sequence performed by bullfinches to master the string pulling task. A) The bird is on the perch where the string is attached and B) grabs the string to C) lift the plastic container. D) The string is maintained in place with the leg and E) the procedure is repeated with lift-grab combinations until F) the container is reachable.

The string-pulling task featured a transparent cylindrical container (height: 3 cm, diameter: 3 cm) in which a food reward (finch seed mix for bullfinches, soaked dog pellets for grackles) was suspended. This container was attached to a wooden perch using a 25 cm (bullfinches) or 50 cm (grackles) string that was suspended inside a transparent PVC cylinder (height: 60 cm, diameter: 6 cm) so that it prevented the birds from obtaining the reward by flying to it (see Fig 1). The task was presented for a maximum of 10 trials of 5 minutes each with 10 min between two trials, on the same day. A bird was considered successful if it pulled the container to its reach and fed from it. The string-pulling test was presented on the 7^th^ day of captivity, without habituation to the task, and after all other behavioral tests were completed (see S1 Appendix). To test for potential improvement in performance, the task was presented again to solvers five minutes after their first success.

We compared performance of our 73 individuals on the string pulling test to data we obtained in previous experiments (see [18,19] and S1 Appendix for details). Here, we briefly summarize the six tasks used in these experiments. All birds were given a two-day period of habituation to captivity after capture. On day 2 (grackles) or 3 (bullfinches), each individual's latency to feed following presentation of an open food dish by an experimenter was measured (shyness test). On day 3, latency to feed from an open dish was again measured, but this time with a novel object placed beside the feeding dish. We measured neophobia as the latency to feed in this test minus the latency to feed in the shyness trial with no novel object. Birds were then given 10 five min trials of a problem-solving task in which they had to flip a lid (grackles) or pull a lid or a drawer (bullfinches) on a transparent box that contained seeds (bullfinches) or a piece of soaked dog pellet (grackles). We started measuring the latency to succeed when an individual touched the apparatus for the first time, thus removing initial boldness or neophobia effects from the problem solving latency. A second problem-solving task, the tunnel task, was given on the next day. It consisted of a transparent rectangular box opened on only one side. A transparent cylindrical tube containing food was inserted at the closed end of the tunnel and a wooden stick was attached to it so that the birds had to pull on the stick to get the tube out of the tunnel. Birds were again given a maximum of 10 trials each lasting 5 min and problem-solving latency was measured in the same way as for the previous task. For bullfinches, discrimination learning was assessed with two petri dishes (same as the one used for shyness) each inserted in a wooden platform painted either green or yellow, open on one side, and placed at each extremity of the cage. The dish inside the unrewarded color contained seeds glued to the bottom of the dish, so that no difference could be seen from a distance but the seeds were impossible to remove for the birds (see [18] and S1 Appendix for details). For grackles, the apparatus consisted of two lid-covered cylinders (same as the lid-flipping task) covered with different colors of tape. Contrary to the lid-flipping task, the birds could not detect the presence of food inside the cylinders because of the opaque tape, but could associate a color with the presence of a reward. The learning criterion was choice of the correct color on seven consecutive trials. Reversal learning on both tasks was assessed the following day by switching the rewarded color and measuring the number of trials to achieve the same success criterion as in the initial discrimination learning phase. Note that the reversal learning test on grackles was not described in Ducatez and colleagues [19] but it is summarized here to compare with string-pulling performance.

### Analyses

We first built models using all behavioral variables. Linear models were built using latency to succeed the string pulling as the dependent variable. Shyness and neophobia latencies, trials to criterion on the discrimination learning and reversal tasks, as well problem-solving latencies were used separately as explanatory variables. We ran two versions of these models, one with and one without non-solvers included in the analyses; in the former case, non-solvers were assigned the maximum latency plus one (3001 seconds).

We then built a second round of linear models that included all the aforementioned variables as well as body condition, weight, sex and capture site as potential confounding variables in the models. We also conducted the analyses with success or failure at string pulling as a binary response variable instead of the latency to succeed, incorporating all explanatory variables mentioned above, using a binomial distribution and a logit link. Stepwise variable selection was achieved using all variables in single models (one model for solvers only and another that included non-solvers). JMP 11.0 software (SAS Institute, Cary, NC) was used to compute all linear models. The p-value threshold was determined using a Bonferroni correction according to the number of models conducted for each category.

## Results

Eighteen out of the 42 bullfinches (43%) succeeded in completing the string-pulling task within 10 trials (mean = 4.2 trials, Std. Dev. = 2.8). Two out of the 31 tested grackles also completed the task (on the first and seventh trials). Typically, the birds that succeeded pulled the string a few times using only their beak, and then started to jointly use their beak and their foot to hold the string and bring the container closer to the perch they were standing on (Fig 1, S1 and S2 Movies). For both species, 3 to 5 pull-grab movements were necessary in order to bring the container close enough to feed from it, and they used the exact same technique when successful. We presented the task a second time to solvers. All of them completed the task during the 5-minute trial following the first success. In bullfinches, mean latency was much lower for the second success than for the first (1^st^: 1135 ± 203.3 s; 2^nd^: 75.29 ± 16.49 s; *p*_t-test_ < 0.001). For the two successful grackles, one was, like the bullfinches, 15 times faster on its second success than on its first (1^st^: 1914 s, 2^nd^: 126 s). The second grackle did not significantly improve, however, possibly due to its very fast performance on its first success (1^st^: 62 s, 2^nd^: 83 s). Most bullfinches gradually learned to master the technique by trying several times to grab the string (Fig 1B and 1C), then implementing grabs using a foot hold (Fig 1D and 1E) and finally coordinating a sufficient number of the latter to master the task (Fig 1F; see S2 Fig for a summary of the mean number of trials required for each movement).

In bullfinches, the latency to succeed was not affected by shyness (solvers: r^2^ = 0.007, *p* = 0.7533; all animals: r^2^ = 0.046, *p* = 0.1789, S1A Fig) or neophobia (solvers: r^2^ = 0.003, *p* = 0.8277; all animals: r^2^ = 0.011, *p* = 0.5379, S1B Fig). Problem-solving scores on the two other tasks performed by the same animals (see [18]) were not associated with string-pulling scores, whether the non-solvers were included in the analysis or not (Tunnel task: solvers: r^2^ = 0.034, *p* = 0.6360, all animals: r^2^ = 0.005, *p* = 0.6691, S1C Fig; Lid-Drawer task: solvers: r^2^ = 0.043, *p* = 0.4068, all animals: r^2^ = 0.024, *p* = 0.3267, S1D Fig). Likewise, string-pulling was not associated with discrimination learning (solvers: r^2^ = 0.001, *p* = 0.8850, all animals: r^2^ = 0.047, *p* = 0.1694, S1E Fig) or reversal learning scores among solvers (r^2^ = 0.163, *p* = 0.0962, S1F Fig, solid line). There was a significant negative correlation between latency to succeed and reversal learning scores when non-solvers were included in the analysis (r^2^ = 0.146, *p* = 0.0125, S1F Fig, dashed line), but with a Bonferroni correction, the result was non-significant (n _TESTS_ = 10; *α* = 0.005). Including sex, body condition and capture site in the previous models did not change any of the results, and these new variables were not significantly associated with string-pulling scores. Building a linear model with success or failure as a response variable and all the aforementioned variables did not yield any significant predictor of success at string pulling following stepwise selection.

Given the low number of successful grackles (2 out of 31), we could not build linear models for this species. The two successful individuals differed sharply in their responses to the behavioral and cognitive tests. One of them was particularly shy and did not eat during the shyness trial. This same individual did not eat either during the neophobia trial. It did not solve any of the problem-solving tasks, but, in contrast, it ranked 6^th^ on the discrimination learning task, reaching the success criterion after 17 trials (mean number of trials for the 31 individuals = 34.22 ± 3.54), and ranked 13^th^ for reversal learning, reaching the success criterion after 47 trials (mean number of trials for the 31 individuals = 56.23 ± 4.44). The second individual behaved very differently: it was relatively bold (it ate after 245 seconds at the shyness trial, ranking 14^th^), not neophobic (it ate after 4 seconds on the neophobia trial, ranking 2^nd^); this individual also solved the two problem-solving tasks (after 2122 s for lid-flipping, as compared to an average of 2165 ± 204 s for the 31 individuals, and 2748 s for the tunnel, compared to an average of 2566 ± 509 s for the 31 individuals). In contrast, it ranked 26^th^ out of 31 individuals at discrimination learning (succeeding after 52 trials), and 19^th^ at reversal learning (succeeding after 54 trials).

## Discussion

Our study provides new information on two species that differ substantially in their ability to acquire the string pulling task. The strong performance of bullfinches is in line with their particularly high innovativeness in Barbados [17]. The Barbados bullfinch is a close relative of Darwin’s finches and belongs to a family, *Thraupidae*, that shows a high propensity for innovative behaviors [17]. The fact that the entire clade of Darwin's finches seems to be innovative led Tebbich and colleagues [24] to propose a version of West-Eberhard's [25] flexible stem hypothesis. Tebbich and colleagues found that woodpecker finch physical cognition did not differ from that of non-tool using Galapagos finches, suggesting that innovativeness could have played a role in the whole ancestral clade's ability to colonize new environments and diversify. The string-pulling literature suggest a similar clade-level effect for *Thraupidae*: Millikan and Bowman [16] concluded that the string-pulling success of woodpecker finches was not superior to that of the four other successful, but non-tool using Galapagos finches or of the tufted titmice the study also tested. The strong performance of Barbados bullfinches further suggests that Thraupid cognitive performance is a general property of the whole clade. Environmental conditions in Barbados (low level of predation and competition, limited resources due to small island size, intense anthropogenic modification of the original environment) are also likely to facilitate the emergence of innovative behaviors. Interestingly, the negative results of Millikan and Bowman [16] on the Cuban grassquit (another Thraupid species), if they are not a false negative due to small sample size, parallel the poor performance on other cognitive tests of the sister species of *T*. *canora* in Barbados, the black-faced grassquit *T*. *bicolor* [17,26].

If the strong performance of Barbados bullfinches here is in line with the generally positive results (five out of six Galapagos Darwin’s finch species tested) that Millikan and Bowman [16] obtained on *Thraupidae*, the much lower numbers of successful Carib grackles also fit with Millikan and Bowman's negative results on the two Icterid species they tested. Given that the number of birds per species that they tested is unspecified in Millikan and Bowman's [16] paper, it is impossible to say whether a large sample of Brewer’s blackbird and Red-winged blackbird could have revealed a low, but nonetheless non-zero number of string-pulling success in these species as it did in Carib grackles. It is ironic that given our much weaker results on grackles compared to bullfinches, one of the very few anecdotal observations of string-pulling in the wild is on the Carib grackle’s sister species, the Greater-Antillean grackle *Q*. *niger*. Graves [27] describes how a grackle used a sequence of beak pulling and foot holding actions to pull up the hind section of a dead anole that was dangling on a piece of skin.

For the first time, to our knowledge, individual variation in string pulling performance was compared to six other behavioral measurements that included temperament, learning and problem-solving. However, despite the diversity of tasks considered in our study, we were not able to link string-pulling performance with either shyness, neophobia, problem-solving or discrimination and reversal learning performance. The only association we found was a *negative* one between reversal learning and string-pulling in bullfinches, but this result was not significant after correction for our large number of comparisons, and was mostly due to a potential outlier (see S1F Fig). In any case, this association was weak, confirming the low, if not inexistent, correlation of performance at string-pulling with other measures. This result was particularly robust in bullfinches, where 18 out of 42 birds succeeded at the string-pulling task, allowing us to run linear analyses. In grackles, only two birds succeeded, precluding statistical analysis, but they were positioned at opposite ends of the speed-accuracy continuum identified in previous work [19]. One grackle was bold and not neophobic, fast at problem solving, but made many learning errors, while the other showed the opposite pattern. These results confirm that, similar to the situation in bullfinches, performance at string-pulling is not correlated with other measures in grackles.

Our results suggest that the string-pulling task may involve skills that differ from the mix of memory, motivation, motor diversity and/or persistence that affect performance in problem solving and in discrimination and reversal learning. Physical cognition, and potentially causal reasoning, might be necessary to succeed at this task, as individuals need to organize behaviours requiring a high number of sequential and coordinated operations. Comparing performance on string-pulling and other physical cognition tasks, such as the cane (e.g. [28,29], trap-tube (e.g. [30]) or water displacement tasks (e.g. [31]), might provide important information on the cognitive skills required to solve the string-pulling task. Differences in physical cognition and causal reasoning abilities might explain the differences in performance we obtained between bullfinches and grackles, in a way problem-solving, discrimination and reversal learning tests could not.

Even if a few of our birds succeeded on the very first trial (two bullfinches and one grackle), most solvers required several trials in which they tried different strategies that gradually led to success (S2 Fig). Furthermore, there was a significant improvement when the task was given a second time to the solvers: latency decreased 15-fold in bullfinches and one of the two successful grackles. Taken together, these data suggest that motor trial-and-error learning was occurring, at least in some individuals. The gradual improvement leading to success over trials and the latency reduction following repeated successes is also seen in the majority of other string-pulling investigations in birds [9,12,15,32,33]. Because our birds were wild-caught, we cannot exclude that differences in individual experience in the field might have affected performance, facilitating for instance the rapid success of the birds that solved in their first trials.

While recent research has focused on modifications of the basic string-pulling procedure we used here to pinpoint the animal's understanding of the task [12,34,35], there has been little work on the relationship between string-pulling and other behavioral and cognitive variables. The fact that we did not detect any correlations in our study is puzzling, despite the fact that previous work on Barbados bullfinches and Carib grackles has found coherent relationships between the measures we tested against string-pulling [18,19]. It is possible that string pulling is associated with traits we did not measure, but Millikan and Bowman [16] had already expressed surprise that tool-using woodpecker finches did not differ from non-tool using Darwin's finches in their ability to solve the test.

One important feature of current research is the use of task variants that manipulate the perceptual and causal cues between the string and the reward [12,34,35]. For example, Taylor and colleagues [12] have found that the New Caledonian crow’s performance was greatly reduced when a platform was used to eliminate the visual feedback of the meat getting closer to the animal. When the same apparatus was employed with a mirror that provided visual feedback, performance was comparable to that of the basic string-pulling paradigm, suggesting that operant conditioning, rather than insight, is the mechanism enabling the birds to master this task. Similarly, when the string is attached to a pulley so that the birds need to pull down in order to get the suspended reward, which is counter-intuitive for the birds, the success rate is also significantly reduced [35]. We do not deal with these issues in our study, so we cannot infer the level of physical cognition involved in our birds' success. The next step would obviously be to use similar protocols with Barbados bullfinches in order to disentangle which mechanisms lead to the high string-pulling success in this species.

## Supporting Information

S1 Appendix Supplementary methods. (DOCX)

S1 Fig Linear regressions between string pulling performance and all other measured behavioral traits. String-pulling latency vs A-B: Shyness and neophobia, C-D: Problem-solving, E-F: Discrimination and reversal learning. Linear regressions with all animals including non-solvers on the string pulling task (which were attributed the maximum latency +1: 3001 s) are represented by dashed lines whereas filled lines are linear regressions in which non-solvers were removed.
(PDF)

S2 Fig String-pulling success progression in Barbados bullfinches. Average number of trials needed for Barbados bullfinches to reach every major step of the string pulling task. Bars represent means ±SEM.
(PDF)

S1 Movie Barbados bullfinch performing string-pulling. (MP4)

S2 Movie Carib grackle performing string-pulling. (MP4)

S1 Table Individual string pulling data. (PDF)
