# Supplementary material for: Bajan Birds Pull Strings: Two Wild Antillean Species Enter the Select Club of String-Pullers
Source: PLoS One. 2016 Aug 17;11(8):e0156112. doi: 10.1371/journal.pone.0156112 (PMC4988674; doi:10.1371/journal.pone.0156112)
Supplement: S1 Appendix — (DOCX) [file pone.0156112.s001.docx]

## Supporting Information: methods

*Capture and sexing.* Barbados bullfinches were caught using mist nets (see Audet, Ducatez, & Lefebvre, 2016 for details) and Carib grackles using baited (with dog pellets) walk-in traps (1 * 0.55 * 0.55 m; see details in Ducatez, Audet, & Lefebvre, 2015). All birds were visually but not acoustically isolated from each other (see Audet et al. 2016 and Ducatez et al. 2015 for more details on captivity conditions). Grackles were sexed based on morphological and behavioral observations, see Overington et al. (2011). Bullfinches were sexed with PCR using blood samples (Audet, Ducatez, & Lefebvre, 2014).

*Behavioral tasks.* The behavioral tasks were different for bullfinches and grackles, as we initially investigated the two species for independent projects with slightly different objectives. In bullfinches, problem-solving abilities were first assessed using a lid-drawer task which consisted of a 2 cm x 3 cm x 3 cm drawer made of white plastic constructed with a circular opening (1.5 cm diameter) at the top covered with a lid to which a hook was attached (Fig. S2C). The birds had the opportunity to gain access to food by opening the lid or by pulling the drawer. Birds were given a maximum of 15 trials each lasting 5 min. The problem solving score was defined as the latency to succeed after the individual touched the apparatus for the first time. The second problem-solving task, the tunnel task, consisted of a transparent rectangular box (H: 3 cm x W: 3 cm x L: 10 cm) opened on only one side (Fig. S2E, Movie S2), and is described in the main text. A tube had to be extracted from the tunnel, and the bird then had to remove the lid to gain access to seeds. Birds were given a maximum of 15 trials each lasting 5 min and problem-solving latency was measured in the same way as for the lid-drawer task.

In grackles, the first problem-solving task (hereafter called lid-flipping task) consisted of a transparent PVC cylinder (diameter = 3 cm, height = 5 cm), set in the middle of a petri dish, over which we placed a white plastic lid (3.4 cm diameter). The visible food reward (1/6 soaked dog pellet) was placed inside the cylinder. To solve the task, a bird had to flip the lid off the cylinder to gain access to the food (see Ducatez et al. 2015). The second problem-solving task (similar to the tunnel task used with bullfinches) consisted of a semi-transparent PVC ‘tunnel’ box open on one end (13 * 4 * 3.5 cm, see Ducatez et al. 2015). An uncovered transparent cylinder (2 cm diameter, 3 cm height) containing the food reward was glued to the end of a wooden stick (17 X 1 cm) equipped with a metal O-ring (which acted as a handle) on the other end. This stick assembly was introduced inside the tunnel so that the transparent cylinder touched the closed end of the tunnel. To solve the task, a bird had to pull the wooden stick out of the tunnel to gain access to the reward. For both tasks, a bird accessing food once was considered as successful, and each individual was allowed a maximum of 10 trials of 5 min each with 10 min between two trials per task.

Associative learning was assessed using a standard color discrimination task, in which the birds had to associate a given color with a food reward. For bullfinches, the test apparatus consisted of two Petri dishes, each inserted in a wooden platform (10 x 10 x 10 cm) painted either green or yellow and open on one side (see main text and Audet et al. 2016). For grackles, the apparatus consisted of two lid-covered cylinders (as in the lid-flipping task) covered with different colors of tape. Note that immediately after the last obstacle-removal trial, we eliminated initial individual differences in motor performance by shaping all birds to the same fast, reliable level of lid-flipping before testing started (see Ducatez et al. 2015 for details). A “color bias” trial was first conducted, where the bird was allowed to eat from one dish/cylinder, and the color chosen by the bird was considered its preferred color. The other color thus became the rewarded one in order to control for initial color bias. The bird was given up to 5 min to choose a dish. If the bird chose the rewarded color, it was allowed to feed (15 sec. for bullfinches, 1/6 soaked dog pellet for grackles). If the bird chose the unrewarded color, the two apparatuses were immediately removed by the experimenter. The location of the rewarded apparatus was switched at each trial to control for spatial preference. The learning criterion was choice of the correct color on seven consecutive trials (Boogert, Monceau, & Lefebvre, 2010). On the next day, the rewarded color was switched and became the non-rewarded color. The same criterion as in the acquisition learning phase was used to establish success. On the first reversal learning trial, all birds initially chose the previously rewarded color (which was incorrect at this stage), indicating that they effectively learned the color stimuli, and not a potential perceptible difference in the Petri dishes.

Audet, J.-N., Ducatez, S., & Lefebvre, L. (2014). Morphological and Molecular Sexing of the Monochromatic Barbados Bullfinch, Loxigilla barbadensis. *Zoological Science*, *31*(10), 687–691. http://doi.org/10.2108/zs140025

Audet, J.-N., Ducatez, S., & Lefebvre, L. (2016). The town bird and the country bird: problem solving and immunocompetence vary with urbanization. *Behavioral Ecology*. http://doi.org/10.1093/beheco/arv201

Boogert, N. J., Monceau, K., & Lefebvre, L. (2010). A field test of behavioural flexibility in Zenaida doves (Zenaida aurita). *Behavioural Processes*, *85*(2), 135–41. http://doi.org/10.1016/j.beproc.2010.06.020

Ducatez, Audet, J. N., & Lefebvre, L. (2015). Problem-solving and learning in Carib grackles: individuals show a consistent speed–accuracy trade-off. *Animal Cognition*, *18*(2), 485–96. http://doi.org/10.1007/s10071-014-0817-1

Overington, S. E., Cauchard, L., Côté, K.-A., & Lefebvre, L. (2011). Innovative foraging behaviour in birds: What characterizes an innovator? *Behavioural Processes*, *87*(3), 274–285. http://doi.org/10.1016/j.beproc.2011.06.002
