# Supplementary figures and images for: Bajan Birds Pull Strings: Two Wild Antillean Species Enter the Select Club of String-Pullers
Source: PLoS One. 2016 Aug 17;11(8):e0156112. doi: 10.1371/journal.pone.0156112 (PMC4988674; doi:10.1371/journal.pone.0156112)

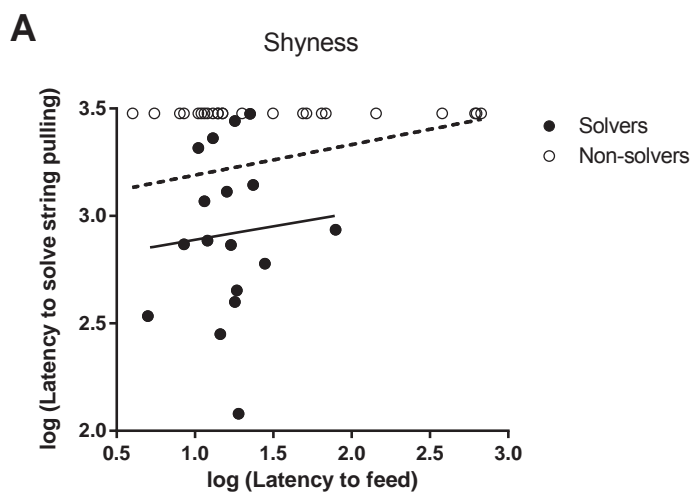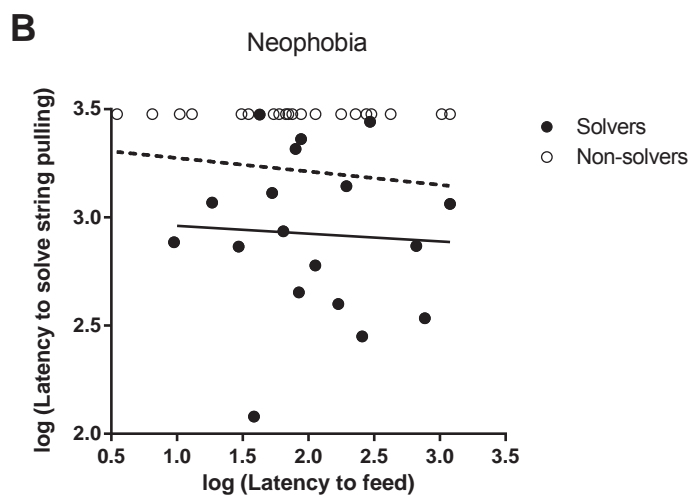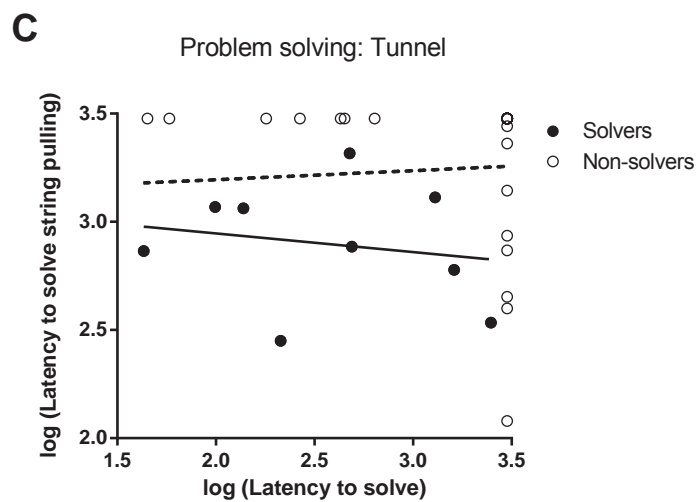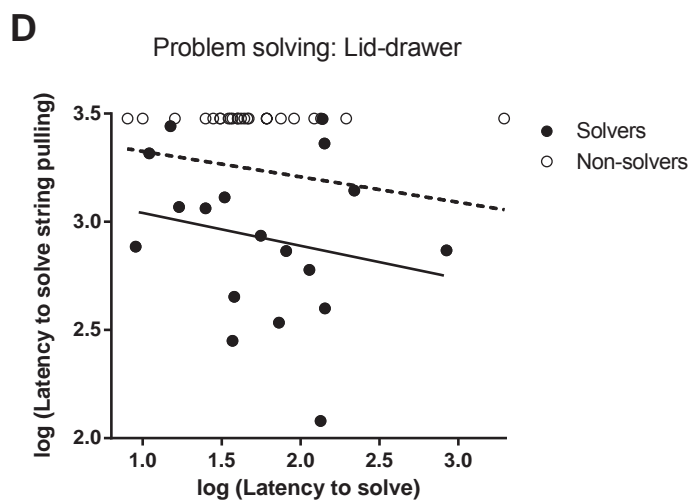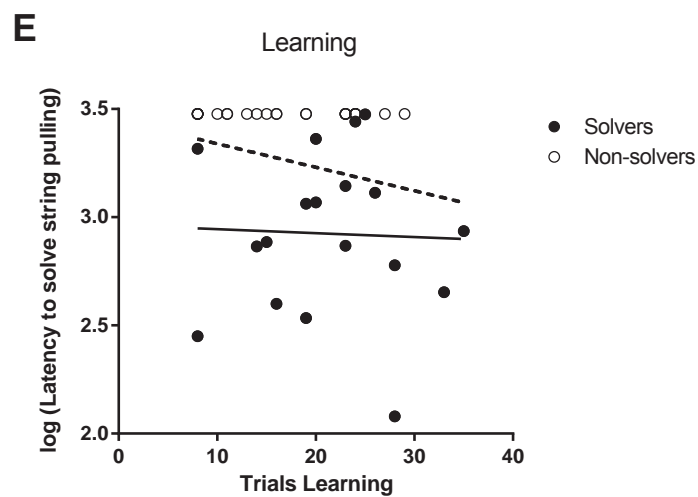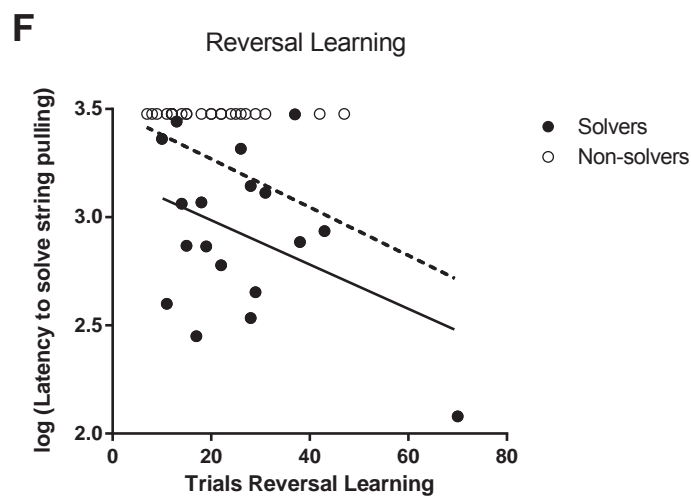

Supplement: S1 Fig — String-pulling latency vs A-B: Shyness and neophobia, C-D: Problem-solving, E-F: Discrimination and reversal learning. Linear regressions with all animals including non-solvers on the string pulling task (which were attributed the maximum latency +1: 3001 s) are represented by dashed lines whereas filled lines are linear regressions in which non-solvers were removed. (PDF) [file pone.0156112.s002.pdf]

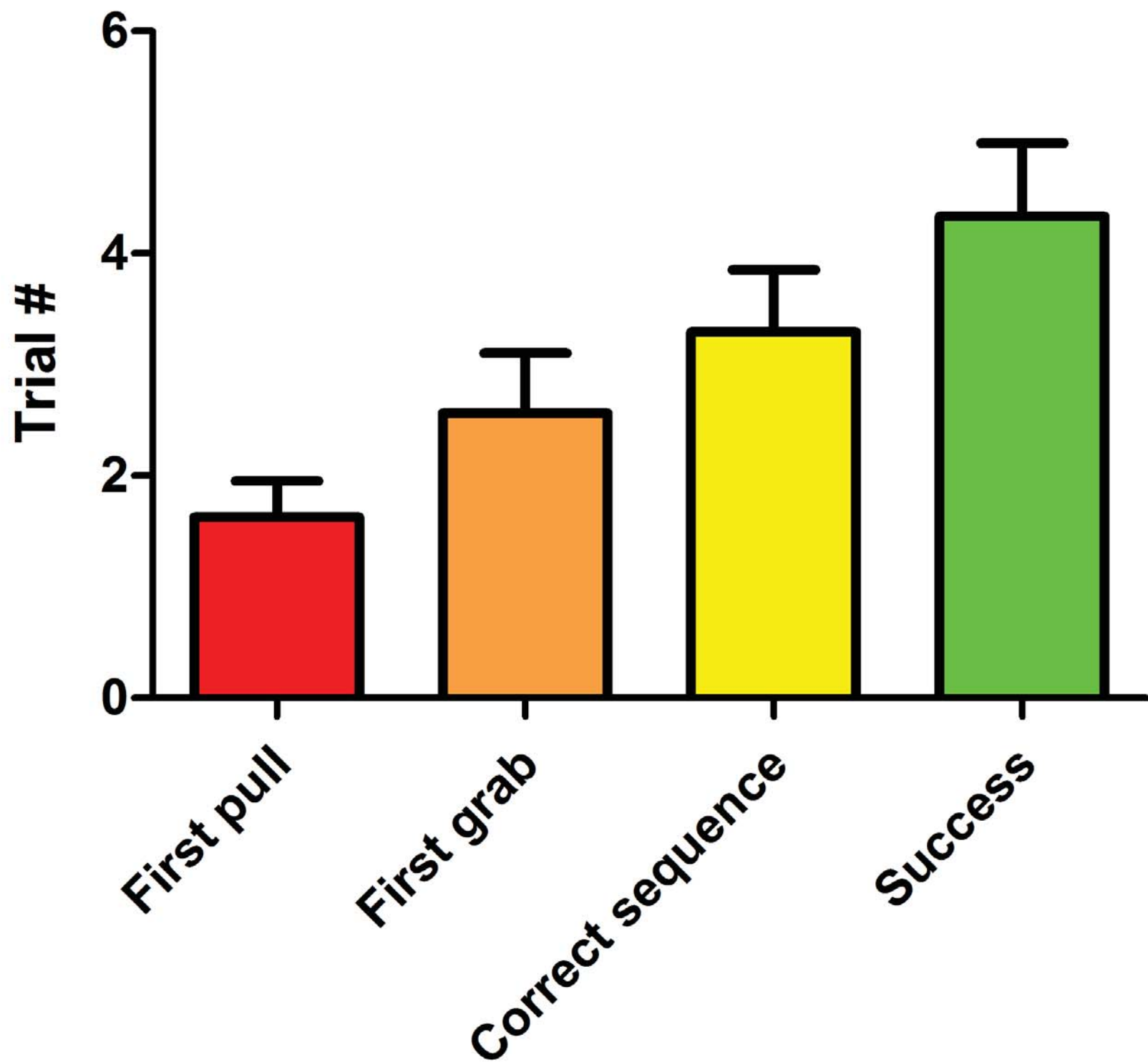

Supplement: S2 Fig — Average number of trials needed for Barbados bullfinches to reach every major step of the string pulling task. Bars represent means ±SEM. (PDF) [file pone.0156112.s003.pdf]
