# Supplementary material for: Bajan Birds Pull Strings: Two Wild Antillean Species Enter the Select Club of String-Pullers
Source: PLoS One. 2016 Aug 17;11(8):e0156112. doi: 10.1371/journal.pone.0156112 (PMC4988674; doi:10.1371/journal.pone.0156112)
Supplement: S1 Table — (PDF) [file pone.0156112.s006.pdf]

Number	Sex	String pulling performance				
		Trials	Latency (s)	Log (latency)	Success/Fail	2nd Latency (s)
A12.O	F	2	342	2.5340	S	62
A14.O	F	5	1395	3.1446	S	59
A16.O	M	11	3001	3.4773	F	NA
A17.O	M	11	3001	3.4773	F	NA
A5.V	M	2	600	2.7782	S	NA
B10.V	M	3	767	2.8848	S	33
B11.V	M	3	732	2.8645	S	30
B12.O	F	11	3001	3.4773	F	NA
B12.V	M	11	3001	3.4773	F	NA
B13.V	M	10	2990	3.4757	S	60
B14.V	F	11	3001	3.4773	F	NA
B15.O	M	1	282	2.4502	S	110
B15.V	F	11	3001	3.4773	F	NA
B16.O	F	11	3001	3.4773	F	NA
B16.V	F	11	3001	3.4773	F	NA
B17.O	M	2	450	2.6532	S	8
B17.V	F	3	862	2.9355	S	105
B18.O	M	4	1154	3.0622	S	15
B18.V	F	11	3001	3.4773	F	NA
B19.O	M	4	1170	3.0682	S	60
B19.V	M	11	3001	3.4773	F	NA
B2.V	M	5	1296	3.1126	S	120
B20.O	M	11	3001	3.4773	F	NA
B23.O	F	11	3001	3.4773	F	NA
B24.O	F	11	3001	3.4773	F	NA
B25.O	F	10	2767	3.4420	S	301
B26.O	F	11	3001	3.4773	F	NA
B27.O	M	11	3001	3.4773	F	NA
B28.O	F	8	2300	3.3617	S	51
B29.O	F	2	398	2.5999	S	119
B3.V	F	11	3001	3.4773	F	NA
B30.O	M	11	3001	3.4773	F	NA
B4.V	M	1	120	2.0792	S	50
B6.V	M	11	3001	3.4773	F	NA
B7.O	F	11	3001	3.4773	F	NA
B7.V	M	11	3001	3.4773	F	NA
B8.O	NA	11	3001	3.4773	F	NA
B8.V	F	11	3001	3.4773	F	NA
B9.V	F	11	3001	3.4773	F	NA
P5.O	M	7	2070	3.3160	S	75
P6.V	F	11	3001	3.4773	F	NA
SP1.V	M	3	737	2.8675	S	22
